# Dietary Polyphenols and Non-Alcoholic Fatty Liver Disease

**DOI:** 10.3390/nu13020494

**Published:** 2021-02-03

**Authors:** Ludovico Abenavoli, Tiziana Larussa, Alessandro Corea, Anna Caterina Procopio, Luigi Boccuto, Marcello Dallio, Alessandro Federico, Francesco Luzza

**Affiliations:** 1 Department of Health Sciences, “Magna Graecia” University, Viale Europa, 88100 Catanzaro, Italy; alessandro.coales@gmail.com (A.C.); procopioannacaterina@gmail.com (A.C.P.); luzza@unicz.it (F.L.); 2 Greenwood Genetic Center, Clemson University, Clemson, SC 29631, USA; lboccut@clemson.edu; 3 Department of Precision Medicine, University of Campania Luigi Vanvitelli, via Pansini 5, 80131 Naples, Italy; marcello.dallio@gmail.com (M.D.); alessandro.federico@unicampania.it (A.F.)

**Keywords:** food, steatosis, antioxidant, insulin-resistance, steatohepatitis

## Abstract

Non-alcoholic fatty liver disease (NAFLD), which is emerging as a major public health issue worldwide, is characterized by a wide spectrum of liver disorders, ranging from simple fat accumulation in hepatocytes, also known as steatosis, to non-alcoholic steatohepatitis (NASH) and cirrhosis. At present, the pharmacological treatment of NAFLD is still debated and dietary strategies for the prevention and the treatment of this condition are strongly considered. Polyphenols are a group of plant-derived compounds whose anti-inflammatory and antioxidant properties are associated with a low prevalence of metabolic diseases, including obesity, hypertension, and insulin resistance. Since inflammation and oxidative stress are the main risk factors involved in the pathogenesis of NAFLD, recent studies suggest that the consumption of polyphenol-rich diets is involved in the prevention and treatment of NAFLD. However, few clinical trials are available on human subjects with NAFLD. Here, we reviewed the emerging existing evidence on the potential use of polyphenols to treat NAFLD. After introducing the physiopathology of NAFLD, we focused on the most investigated phenolic compounds in the setting of NAFLD and described their potential benefits, starting from basic science studies to animal models and human trials.

## 1. Introduction

Non-Alcoholic Fatty Liver Disease (NAFLD) is a clinical condition primarily characterized by fat accumulation in liver parenchyma (>5% of hepatocytes). Its clinical burden is encompassed in its pathological spectrum, which ranges from simple fatty liver (Simple Fatty Liver—SFL) to steatohepatitis (Non-Alcoholic Steatohepatitis—NASH), ending with hepatic cirrhosis and hepatocarcinoma, through a progressive fibrosis of the organ [1]. These advanced stages are associated with higher mortality, but all stages of NAFLD can significantly increase the risk of cardiovascular diseases, since these are the most prevalent clinical features in NAFLD [2]. Its definition accounts for a condition in which fat in liver parenchyma is above the normal quantity and shows the same histopathological features of alcohol-induced liver steatosis, but it is recognized in patients who drink little to no alcohol [3]. The histopathological hallmark of NAFLD is steatosis, the common central feature, which is the accumulation of lipid droplets in hepatocytes; signs of cell damage, such as ballooning and apoptotic changes and Mallory-Denk bodies are also typical, while the portal and lobular inflammatory infiltrate is more specific tothe NASH stage [3].

The international threshold observed by international guidelines to distinguish the alcohol-induced fatty liver disease from NAFLD is represented by two drinks, corresponding to 20 g of alcohol intake per day [4].

NAFLD is the most common chronic liver disease worldwide and its prevalence is constantly rising in the global population, concurring with that of diabetes and obesity. Twenty-five percent of adults are affected by NAFLD and its prevalence doubles in diabetic or obese subjects [5,6]. An increase of NAFLD incidence was reported in the last two decades, not only in Western countries, where a sedentary lifestyle and obesity are strongly present in association with hyper-caloric diet, but also in urban areas of developing countries [7,8]. Approximately a third of patients with SFL eventually develop NASH, although most of them remain asymptomatic [5]. The uprising incidence and the clinical burden of most advanced stages of NAFLD, i.e., complicated cirrhosis, hepatocarcinoma, or ischemic heart disease, make the development of epidemiological, behavioral, and effective therapeutic strategies a high priority [9]. In the last years, several therapeutic options were proposed to treat NAFLD. However, the various approaches so far proved insufficient forimproving liver metabolic function and pathophysiological features of hepatocyte stress and inflammation [10]. Approved treatments for NAFLD still do not exist and the available options are essentially based on counsel to switch to healthy lifestyle behavior, such as a healthy diet, low in fats and carbohydrates, and daily physical activity [11,12].

Polyphenols are a diversified class of vegetable-derived compounds sharing the chemical property of being hydrosoluble [13]. They are widely found in fruits, tea, red berries, coffee, red wine, and dark chocolate; are well-known as antioxidant agents, and wereproposed as a treatment for several metabolic disorders [14]. Polyphenols represent the most abundant antioxidant compounds in the human diet, and their effects, such as those of vitamins, are the cornerstone of the traditionally known benefit of fruits and vegetables in several diseases [13,14]. Studies showed that polyphenols can prevent oxidative stress, promoting fatty acid beta-oxidation, and modulating insulin-resistance [15,16]. Furthermore, it was reported that these compounds might modulate de novo lipogenesis, by acting on the activity of lipogenic enzymes, and improving the expression of lipolytic proteins [17]. 

Thus, throughout the years, several authors aimed to evaluate the effect of polyphenols on metabolic pathologies, such as insulin resistance and NAFLD. This review aims to explore the promising role of polyphenols in treating NAFLD, according to the most recent updates. After introducing the physiopathology of NAFLD, we focused on the most studied phenolic compounds and described their potential clinical benefits in the setting of NAFLD, through the examination of basic science studies, animal experimental models, and studies on human subjects.

## 2. Non-Alcoholic Fatty Liver Disease Pathophysiology

**Figure 1:**
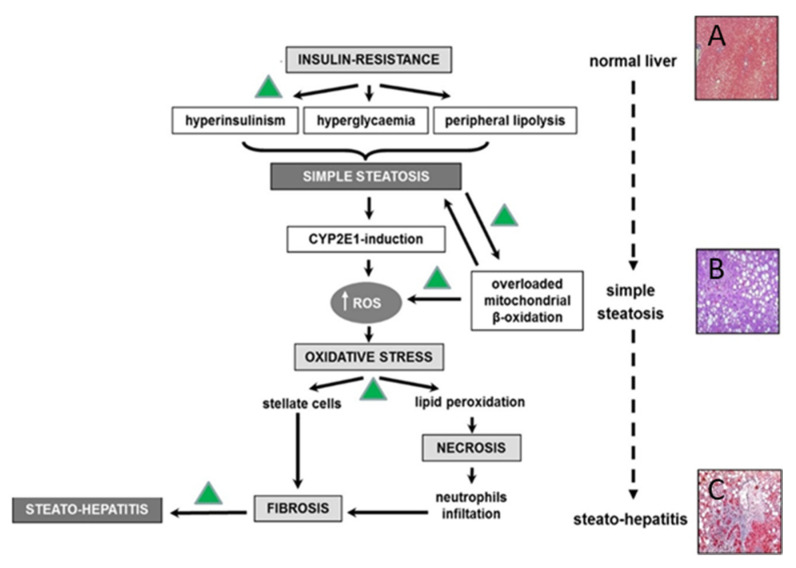
Pathogenic mechanisms involved in the progression of NAFLD and possible sites of polyphenols action (green triangles). (CYP2E1: Cytochrome P450 2E1, ROS: reactive oxygen species). (**A**)Normal liver parenchyma is composed of small lobules of hexagonal shape with portal tracts at the apices; (**B**) hepatocytes contain one or more large fat droplets that displace the nucleus to an eccentric position; and(**C**) ballooning degeneration of hepatocytes, scattered inflammation, and apoptotic bodies.

Pathophysiological mechanisms underlying NAFLD are classically explained by the two hits hypothesis, in which two harmful events in the sequence occur and compromise the function and structure of the liver parenchyma—the accumulation of fatty acids in the liver (the already mentioned steatosis), which represents the first one, and the progressive onset of oxidative stress and hepatocyte damage subsequently [1,12]. This classic scheme is actually considered obsolete and outdated by the concept of speculating an action of more hits acting in parallel and in particular insulin-resistance, oxidative stress, genetic and epigenetic factors, intestinal microbiota, and environmental elements, among others (Figure 1). The association between NAFLD, obesity, diabetes mellitus type 2 (T2DM), and dyslipidemia might suggest that NAFLD is a condition that involves not only the liver but the entire metabolic setting of the body and is influenced by such metabolic setting as well [18]. Hereafter, there is strong agreement that NAFLD is the hepatic border of the metabolic syndrome, well-characterized by metabolic derangement [19]. 

Liver steatosis is the biochemical result of an imbalance between fatty acid uptake and synthesis in the hepatocytes, and their discharge from the cell through beta-oxidation and secretion by Very Low-Density Lipoproteins (VLDLs). In greater detail, the metabolic steps that lead to the accumulation of triglycerides in the hepatocytes are fatty acid uptake, de novolipidogenesis, fat oxidation, and VLDL export into the blood [18,20]. Several enzymes are responsible for lipid metabolism processes—the rate of de novolipidogenesis depends on the activity of mitochondrial Citrate Carrier (CiC), Acetyl-CoA Carboxylase (ACC), Fatty Acid Synthase (FAS), Diacylglycerol Acyltransferase (DGAT).It also depends on transcriptional factors, namely, the Steroid Regulatory Element Binding Proteins (SREBPs), Carbohydrate Element Response Binding Protein (ChREBP), Liver X receptor alfa (LXR-α), Farnesoid X Receptor (FXR), and Peroxisome Proliferator-Activated Receptors (PPARs) [21]. An important emerging role for the pathophysiology of NAFLD is played by mitochondria, which are involved not only in lipid metabolism but also in the setting of oxidative stress increase, pro-inflammatory cytokines production, and insulin-resistance pathogenesis [22]. Mitochondrial membrane damage, hepatocyte apoptosis, inflammatory infiltration, and necrosis are altogether expressions of a pathological continuum occurring in the state of NAFLD/NASH, where the main intracellular actor is the mitochondrion. Furthermore, mitochondria dysfunction is a consequence of all these events, in the context of a vicious circle that overloads the normal activity of the remaining mitochondria [23]. Some evidence shows that oxidative stress is one of the main drivers of hepatocyte damage and contributes actively to the pathological progression from SFL to steatohepatitis [10].

It is important to underline that the diagnosis of NAFLD, to be sensitive and accurate, needs a liver biopsy and histological staining, as histopathology allows clinicians and researchers to score NAFLD activity. This tool is limited in clinical practice by its invasive nature [24]. Hence, researchers focused on less invasive methods to perform studies on NAFLD and to evaluate treatment efficacy [25]. 

## 3. Chemistry of Dietary Polyphenols

**Figure 2:**
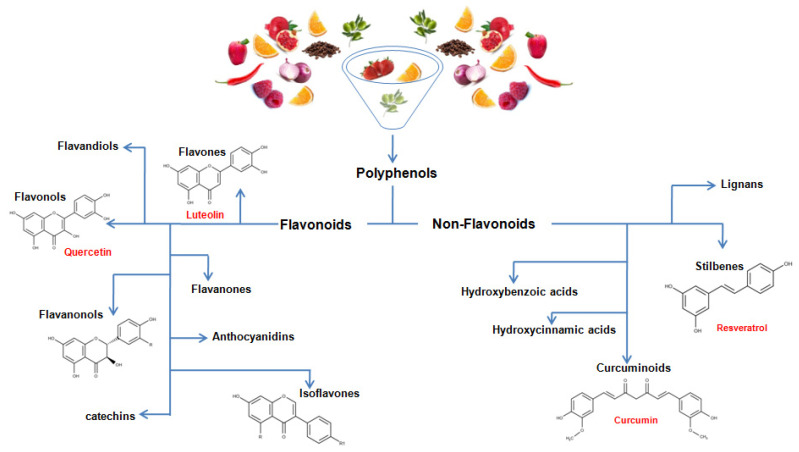
Chemical classification of polyphenols.

**Figure 3:**
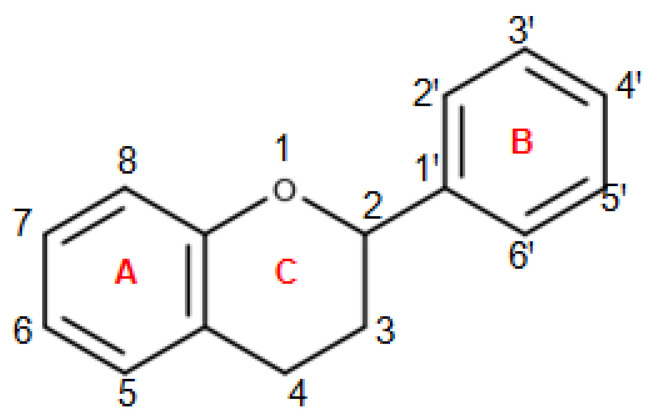
Representation of the chemical structure of 2-Phenylchroman, shows the basicnucleus of flavonoids, indicating the chroman (**A**,**C**) and the aromatic ring in position 2 (**B**).

Polyphenols represent an immense family of organic compounds widely present in natural products. The term polyphenol derives from the presence in the chemical structures of phenolic groups that are established in more or less complex structures. Due to their great variety, polyphenols are classified into flavonoids and non-flavonoids (Figure 2). Among the flavonoids, which represent the largest family of polyphenols, we find flavones, flavandiols, flavonols, flavanonols, catechins, flavanones, anthocyanidins, and isoflavones. Among the non-flavonoids, we find stilbenes, lignans, and phenolic acids, which are divided into hydroxybenzoic acids and hydroxycinnamic acids [26]. Flavonoids are characterized by a basic chemical structure with 15 carbon atoms C6-C3-C6 in which we find two aromatic rings indicated with A and B joined to a pyran indicated byC, as shown in Figure 3. The various classes of flavonoids differ from each other for the degree of oxidation of the C ring and the substitutions of the A, B, and C rings [26]. In this regard, flavones, flavonols, flavanols, and flavanones are characterized by the presence of a carbonyl function on the C4 and by the presence of the aromatic ring B bound to C2, these compounds differ in the substituents present on the rings. Among these compounds, we find quercetin, one of the most famous polyphenols belonging to the group of flavonolsthat are widely spread, for example in red onion, purple potatoes, and peppers. On the contrary, the group of catechins is characterized by the absence of the carbonyl function in C4 and the double bond in C2-C3. Furthermore, the catechins have a hydroxyl group in position C3, which together with the orientation of the benzene ring in C2 are responsible for the configuration of the catechins themselves. The anthocyanidins instead represent cationic molecules characterized by the absence of the carbonyl function in C4 and by two double bonds in C1-C2 and C3-C4. Furthermore, unlike the other flavonoid compounds, isoflavones do not have the typical structure of 2-phenyl-benzopyrone, instead they are characterized by a 3-phenylchromone structure. While a basic chemical structure with common characteristics is recognized in the flavonoids, the group of non-flavonoids represents, on the one hand, a much more heterogeneous cluster. Among the most interesting compounds belonging to non-flavonoids we find resveratrol, belonging to the group of stilbenes, a compound typically present in red wine, characterized by the presence of two aromatic rings spaced by an ethylene chain. Furthermore, in the context of non-flavonoids, one of the noteworthy compounds is certainly curcumin, a curcuminoid characterized by two aromatic rings spaced by an heptandiene chain characterized by two carbonyl functions.

## 4. In Vitro Studies

Studies based on cell cultures represent a well-established tool to directly assess the molecular mechanisms of action of polyphenols in the prevention or treatment of steatosis. Primary cultures of human hepatocytes are the optimal model to perform in vitro research for discovering NAFLD-related mechanisms, but poor liver sample availability is a limitation of this technique [27]. The human hepatocyte-derived cell line HepG2 represents the most used alternative [28]. Several conditions that mimic in vivo NAFLD were validated, such as the treatment of cells with palmitic acid (16:0) or glucose, to induce lipid accumulation and subsequent cell damage in an analogue manner, like NAFLD. Vidyashankar et al. used oleic acid (18:1n-9) to perform their experiments in HepG2 cell cultures and demonstrated that 10 mM of quercetin, a flavonol, reduced TAG accumulation in cells, DNA fragmentation, and inflammatory cytokines TNF-α and IL-8. The proposed molecular mechanisms were the inhibition of lipogenesis and improvement of FA catabolism [29]. In a similar way, Rafiei et al. observed that several pure polyphenols (e.g., quercetin, resveratrol, kuromanin, berberin, catechin, cyanidin) were effective forconferring strong protection to HepG2 cells from oleic acid-induced steatosis, and some of them protected against mitochondrial dysfunction and from aerobic metabolism dysfunction [30]. Itwas found that berberin and kuromanin might act through the modulation of lipidic metabolism, differing from other polyphenols for having a null effect on mitochondria bioenergetics. In an interesting study comprising inpart work on an animal model and inpart on cell cultures, Yan et al. evaluated the effects of curcumin on hepatocyte metabolism [31]. In this model, palmitic acid-induced steatosis compromised xenobiotics and endogenous metabolism of C57BL/6 mice liver cell cultures, affecting the function of CYP3A and CYP7A cytochromes and inducing SREBPs activity. Curcumin treatment effectively reversed these damages, probably due to its ability to regulate metabolism through CYP3A andCYP7A modulation. Furthermore, curcumin increases the expression of Nfr2, FXR, and SHP in rat cells, in order to lower the expression of SREBP1-c e FAS. In this context, liver X receptor α (LXRα), a transcription factor involved in lipid metabolism regulation, seemed to be the key point, and SREBP1-c was proven to be a strong driver of de novo lipogenesis [31]. The pivotal role of SREBP was previously reported by Liu et al. [32]. The authors showed that luteolin was effective in inducing a reduction of palmitate-stimulated lipid accumulation in HepG2 cells, associated with decreased SREBP-1c and FAS gene expression. Diminished activity of ACC was also determined by luteolin. As well as SREBP, FAS is a strong promoter of de novo lipogenesis. ACC is involved in lipogenesis by mediating the conversion of acetyl-CoA into palmitate that subsequently is esterified into TAG in the liver, connecting metabolic pathways of Krebs cycle and lipogenesis. Recently, Khalil et al. tested two formulations of *Thymbra spicata* polyphenol extracts to assay their antisteatotic and antioxidant properties in vitro [33]. Results showed thatboth extracts ameliorated intracellular lipid accumulation, oxidative stress, and inflammation in NAFLD cellular models. The aqueous extract was more effective inreducing hepatic steatosis, while the ethanolic extract had a higher antioxidant potential and wound healing activity [33]. More recently, the role of the Nrf2 signaling pathway, known for its aberrant methylation in cancer pathology, was investigated in an NAFLD cell model of HepG2 cells induced with high glucose concentration. The beneficial effect of resveratrol was evaluated in this setting.Results highlighted that treatment of HepG2 cells with high doses of glucose enhanced the methylation level of the Nrf2 promoter, whereas resveratrol reversed this effect. Furthermore, an in vivo model showed how methylation of the Nrf2 promoter was significantly associated with triglycerides accumulation in the liver and expression of the lipogenic enzymes SREBP and FAS [34]. Different polyphenols, like resveratrol and curcumin, exert their effect through analogue molecular targets both acting on the Nrf2 pathway, suggesting that the same molecular pathways might be shared among these compounds in their lipidic metabolism effect [31]. On this basis, the consumption of antioxidant-rich foods in general, and in particular of foods rich in polyphenols, could be considered as a potential new approach in the treatment of NAFLD and therefore deserves future clinical investigation [11,35].

## 5. Results from Animal Models

All polyphenols or polyphenol-rich extracts reported some efficacy in reducing triglycerides accumulation in the liver, but each tested compound could show a peculiar molecular target. The mechanisms of action ranged from lipidogenesis regulation to modulation of insulin-resistance, oxidative stress modification, and inflammation control [10].

Inflammation and the subsequent change in the inflammasome pathway, with the derangement of cytokines profile, is the focal point for the transition from the simple accumulation of lipids in the liver to NASH. As several pre-clinical studies reported that NAFLD is characterized by histological and biochemical inflammation, polyphenols can ameliorate the inflammatory response and reduce fat accumulation in hepatocytes [36]. Preferred animal models of NAFLD are mice and rats, in which NAFLD is induced by dietary or pharmacological manipulations. Animal models differ in terms of NAFLD phenotype and metabolic characteristics, and some of them are more like human NAFLD than others [37]. For example, the methionine-choline deficient model (MCD) produces a more severe phenotype of NAFLD, with less weight gain but a worse inflammation and oxidative stress, in comparison toa High-Fat Diet (HFD) model [38]. 

Nrf2 was previously recognized as a key regulator of cellular oxidative status balance, with a protective role, and its mRNA is reduced during High Fat-High Fructose (HFHFr)-induced NAFLD [39]. The beneficial effects of curcumin were investigated in a model of NAFLD-induced male rats, with a focus on the role of the Nrf2-FXR-LXRα pathway [31]. It is recognized that LXRα regulates lipid biosynthesis in the liver, through its target gene SREBP-1c. Curcumin was able to lower plasmatic lipid levels and to modify lipid metabolism in C57BL/6 male mice with HFHFr-induced NAFLD, but is also capable ofmodifying the activity of cytochromes CYP3A and CYP7A in vitro and in healthy rat models. Lipid droplet accumulation and fatty acid biosynthesis in the liver were decreased after curcumin treatment, while the reduction of Nrf2 induced by the HFHFr diet was reversed [31]. 

Bergamot is a citrus fruit that grows typically in the Calabria region of Southern Italy and its polyphenol extract, the Bergamot Polyphenol Fraction (BPF), was studied on NAFLD induced by a high-calorie diet in male Wistar rats. The results showed that BPF significantly boosted lipid droplet clearance from the liver and reduced the plasma TAGs. Moreover, BPF improved insulin-sensitivity and modulated hepatic inflammation by reducing the pro-inflammatory cytokine IL-6 and increasing the anti-inflammatory cytokine IL-10 levels [40].

Resveratrol is a polyphenolic compound thatwas proven to be effective in ameliorating liver pathologies [41]. In a mouse model of NAFLD, resveratrol reverted hepatic disfunction associated with nesfatin-1 and glicolipidic metabolism, as showed by the blood levels of transaminases, total bilirubin, total cholesterol, LDL-cholesterol, glycemia, insulinemia, and nesfatin-1. Resveratrol effects in this study were compared with those of other known active drugs used in the treatment of diabetes and insulin resistance, respectively, namely sitagliptin and rosiglitazone. Of note, resveratrol improved the histological degree of steatosis and ameliorated the behavioral and cognitive impairments induced by NAFLD [42]. Another study on the mouse model of HFD reported that resveratrol is capable of preventing liver fat accumulation by enhancing fatty-acid β-oxidation and reducing lipogenesis. In this setting, the central role of AMPK regulation as a modulatory protein of lipid metabolism emerged [43]. Indeed, AMPK is often reported as an interesting molecular target in the setting of NAFLD treatment. In 2008, a cornerstone study byBujanda et al. reported resveratrol as a booster for antioxidant enzymes and in particular catalase, superoxide dismutase, and glutathione peroxidase, in an NAFLD rat model [44].

Green Tea Polyphenols (GTP) are known for the beneficial effects on metabolic syndrome, of which NAFLD is a common hepatic manifestation. In rats, GTP significantly reduced transaminases and fasting glucose blood levels, insulin resistance, and hepatic lipid content. Furthermore, GTP reduced inflammation by decreasing levels of IL-6 and TNF-α and the histopathological hallmarks of liver injury, while increasing the concentration of the antioxidant enzyme superoxide dismutase. The proposed mechanism could be the modulation of AMPK activity, as GTP increased AMPK phosphorylation [45]. 

Chinese raw bowl tea, which is rich in polyphenols, showed benefits in decreasing body weight and liver weight, in HFD-induced NAFLD mice. Moreover, the tea decreased inflammatory cytokines and reactive oxygen species production in the liver, ameliorating the status of liver impairment [46]. Raw bowl tea was able to improve the intestinal environment, positively modifying the gut microbiota, which isstrongly involved in NAFLD alterations and promotion of obesity [39,47]. 

## 6. Clinical Applications

Clinical trials conducted on human subjects suffer from the practical limitation of obtaining liver biopsies to replicate the assessments conducted in studies, using animal models. Hence, surrogate tools for the evaluation of NAFLD are used in this setting, such as surrogate scores, ultrasonography, or magnetic resonance spectroscopy [48,49]. Several trials were conducted with the aim ofevaluating the effects of polyphenols and NAFLD, and the most relevant are the blind, randomized, and placebo-controlled trials (RCTs) [50].

Curcumin was administered in patients with ultrasonographic evidence of NAFLD, 1000 mg × day for 8 weeks, showing a significant reduction in body mass index (BMI), a significant reduction in liver fat content and a reduction in HbA1c and blood glucose levels in curcumin, compared tothe placebo group. A decrease in liver volume and improvement of portal flux in the curcumin group was also shown. Of note, curcumin was safe and well tolerated by subjects [51,52]. 

The biochemical and physiological effects of resveratrol and its benefit on NAFLD was evaluated by a 12-weeks, double-blind, RCT, at a dosage of 75 mg in two oral daily doses. Hepatic fat content was assayed by magnetic resonance spectroscopy. Liver fat content in the resveratrol supplementation group was significantly reduced as compared tothe placebo group, suggesting that resveratrol could prevent the liver fat increase. Resveratrol resulted wassafe and well tolerated during the trial [53]. Another RCT conducted in NAFLD subjects reported that resveratrol significantly decreased aspartate aminotransferase, glucose, and low-density lipoprotein cholesterol, as compared to the placebo group, promoting a role for resveratrol supplementation in treating insulin resistance and its consequences [54]. Accordingly, a 12-weeks trial with 500 mg resveratrol supplementation in 50 patients with NAFLD reduced alanine aminotransferase and hepatic steatosis significantly more than placebo [55].

It is recognized that cardiovascular complications are the major cause of mortality in NAFLD, therefore, the effects of resveratrol on the atherogenic risk factors in NAFLD patients were investigated [56]. Although resveratrol supplementation reduced BMI and waist circumference compared to the placebo group, no significant changes were found in lipid profile, serum atherogenic indices, liver enzymes, and blood pressure. Further evidence is needed to support the efficacy of resveratrol in the management of NAFLD [57].

Silymarin is a complex mixture of 6 major flavonolignans and other minor polyphenolic compounds derived from the milk thistle plant *Silybum marianum*, whose beneficial effects as an antioxidant was reported in patients with NAFLD [58]. In patients with biopsy-proven NASH, silymarin improved fibrosis and liver stiffness.As curcumin and resveratrol, silymarin was found to be safe and welltolerated [59]. Our group reported that an antioxidant complex supplementation including silymarin, associated with physical activity and a healthy diet, is effective inimproving anthropometric parameters, insulin sensitivity, lipid profile, and reducing hepatic fat accumulation and liver stiffness in NAFLD patients [60].

## 7. Conclusions

The interest in polyphenols as nutraceutical supplementation in NAFLD is increasing, as any of these compounds might offer healthy properties for the liver. Considering that NAFLD is an upcoming challenge for scholars and health systems worldwide, it is plausible to consider polyphenols as a potential new therapeutic approach to treat hepatic fat accumulation and its sequelae. This approach is supported by several in vitro studies, animal models, and few clinical trials. Additionally, pre-clinical and clinical settings showedmany effects of polyphenols, likethe increase of fatty acid oxidation and the modulation of insulin resistance, oxidative stress, and inflammation, which represent the main pathogenetic steps of the onset and progression from SFL to NASH. However, data from clinical studies are still limited and often conflicting. Further investigations are needed, especiallythrough randomized clinical trials, for validating the intriguing role of polyphenols in the treatment of NAFLD.
